# The Meta-Analysis of Bevacizumab Combined with Platinum-Based Treatment of Malignant Pleural Effusions by Thoracic Perfusion

**DOI:** 10.1155/2022/1476038

**Published:** 2022-02-25

**Authors:** Bairu Shen, Minghua Tan, Zhengyu Wang, Changshan Song, Hui Hu, Shunfu Deng, Yuxin Yang

**Affiliations:** Department of Thoracic Surgery, Foshan Clinical Medical School of Guangzhou University of Chinese Medicine, Guangzhou, Guangdong, China

## Abstract

**Objective:**

To evaluate the safety of bevacizumab combined with platinum-based thoracic perfusion for treating lung cancer-related malignant pleural effusion (MPE) through meta-analysis.

**Methods:**

The CNKI, PubMed, Cochrane Library, Embase, Chinese Science and Technology Journal Database (VIP), and Wanfang Databases were searched for randomized controlled trials (RCTs) of bevacizumab combined with platinum-based thoracic perfusion for the treatment of MPE. The references included in the articles were manually searched for additional studies. A meta-analysis of the RCTs was conducted using the RevMan 5.3 application.

**Results:**

A total of 8 studies involving 540 patients (271 cases in the test group and 269 cases in the control group) were included in the meta-analysis. The test group had a significantly greater risk of elevated blood pressure as well as a higher rate of complete remission (CR) compared to the control group (*P* < 0.05). In contrast, the incidence of partial remission (PR) was only slightly higher in the test group (*P* > 0.05), and the risks of leukopenia, vomiting or nausea, rhinorrhea, diarrhea, gastrointestinal bleeding or hemoptysis, proteinuria, abnormal kidney and liver function, arrhythmia, and rashes were not significantly different between the test and control groups (*P* > 0.05).

**Conclusion:**

Bevacizumab combined with platinum-based thoracic perfusion can achieve CR of MPE in patients with advanced lung cancer without significantly increasing the risk of adverse effects. The rate of PR was similar for the combination treatment and platinum-based infusion.

## 1. Introduction

The presence of malignant pleural effusion (MPE) in patients with advanced lung cancer is mainly due to cancer cell infiltration or metastasis into the pleura. The molecular basis of MPE pathogenesis is not completely clear, although overexpression of immune-related factors and vascular permeability regulators has been implicated [1]. MPE seriously affects the quality of life, and the median survival of lung cancer patients with MPE is about six months [2, 3]. Currently, advanced lung cancer complicated with MPE is primarily treated with systemic drug therapy and local treatment of the chest cavity. Intraluminal drainage combined with intraluminal injection is the most common local treatment modality; selecting an effective intracavitary can increase therapeutic efficacy with fewer complications [4–7]. Combined intrathoracic perfusion therapy is also an effective treatment modality for MPE, and several studies have reported better outcomes and lower drug toxicity with this approach. However, there are also reports of potential side effects of local perfusion, especially when two groups of drugs are combined. Since the pleural absorption kinetics of drugs differ significantly compared to that of intravenous administration, there are concerns regarding the safety of two-drug perfusion therapy. We conducted a meta-analysis to analyze the safety and efficacy of the combination of bevacizumab and platinum-based thoracic infusion in the treatment of lung cancer-associated MPE.

## 2. Data and Methods

### 2.1. Case Exclusion Standards and Inclusion

#### 2.1.1. Study Type

This is a published Phase III RCT.

#### 2.1.2. Study Subjects

Patients with pathologically or cytologically confirmed advanced non-small-cell lung cancer (NSCLC) or small-cell lung cancer (SCLC)-associated MPE.

#### 2.1.3. Interventions

The RCT group was treated with bevacizumab in combination with platinum-based thoracic perfusion, and the control group was treated with platinum-based thoracic perfusion alone.

#### 2.1.4. Outcome Indicators

Efficacy evaluation: the efficacy was determined according to the WHO evaluation criteria [8]. Complete remission (CR) was defined as the disappearance of pleural effusion for over four weeks, partial remission (PR) as the significant reduction in the volume of pleural fluid by at least 50% for over four weeks, no significant remission (NC) as less than 50% reduction in pleural fluid or no significant change, and progressive disease (PD) as a significant increase in pleural fluid volume and worsening of symptoms after treatment [8]. The patients in each treatment and dosage group were monitored for adverse reactions according to the National Cancer Institute-Common Toxicity Criteria (NCI-CTC) [8].

#### 2.1.5. Exclusion Criteria

(1) Phase I and II RCT studies, (2) reviews or case-control studies, and non-RCT studies such as retrospective cohort studies, (3) concurrent radiotherapy as first-line therapy, (5) incomplete data and unclear study indices, and (6) published in languages other than English and Chinese [9].

### 2.2. Literature Search Strategy

The Wanfang databases, Cochrane Library, Embase, CNKI, PubMed, and VIP databases were systematically searched for RCTs conducted on the safety and efficacy of bevacizumab in combination with platinum for treating lung cancer-related MPE. The search was limited to articles published till December 31, 2019. The keywords used for searching articles published in English included Bevacizumab, Avastin, lung cancer, cisplatin, and carboplatin, whereas the search terms for Chinese literature included Bevacizumab, Avastin, lung cancer, MPE, cisplatin, and carboplatin. The search terms for interventions (“cisplatin or carboplatin,” “Bevacizumab or Avastin,” “thoracic perfusion,” “thoracic perfusion,” “Bevacizumab or Avastin”) were combined with related diseases (“lung cancer and MPE,” “lung cancer and MPE”) using Boolean logic. The references included in each study were manually retrieved to expand the search. In addition, the ProQuest and CNKI platforms were also screened to collect abstracts of dissertations or scientific conferences. Journals in relevant specialized fields were supplemented with manual or other searches to avoid missing relevant literature [9].

### 2.3. Literature Screening and Data Extraction

The literature was reviewed independently by two researchers, and the decision to include any study was made on the basis of the review results. In case of any disagreement, a third researcher was consulted, and the final decision was made through a joint discussion among all three reviewers. The data were extracted from the studies by two researchers independently and cross-checked. Any inconsistencies in the data were resolved by discussing them with a third researcher. The following data were included in the meta-analysis: (i) writer ranking, (ii) publication year, (iii) country of publication, (iv) histological type, (v) the total number of studies, (vi) median age, (vii) treatment regimen, (viii) number of evaluable indicators, and (ix) outcome indicators such as treatment efficacy and complications [9].

### 2.4. Risk of Bias Assessment

The risk of bias was evaluated by applying the Bias Assessment Tool's Cochrane Risk, which includes (i) random sequence generation (selective bias), (ii) allocation concealment (selective bias), (iii) implementation of blinding (implementation bias), (iv) blinded assessment results (measurement bias), (v) completeness of data (missing visit bias), (vi) selective reporting (reporting bias), and (vii) other bias (issues that could clearly lead to a risk of bias, e.g., apparent benefit and early discontinuation of the trial). The risk of bias was classified as low, unclear, and high [9].

### 2.5. Statistical Analysis

Meta-analysis was conducted using the RevMan 5.3 application, with a relative risk (OR) as the outcome. The results were presented using 95% confidence intervals (95% CI), and *P* < 0.05 was set as a statistically significant difference. The heterogeneity between the included studies was analyzed using the *Q* test and quantified using the *I*^2^ index. The fixed-effects model was used in case of low heterogeneity (*P* > 0.05, *I*^2^ < 40%) [9]; otherwise, the random-effects model was applied.

### 2.6. Evaluation of Publication Bias

Publication bias was assessed based on the symmetry of outcome indicators using a funnel plot. A symmetrical funnel plot indicated a lack of any publication bias, whereas an asymmetrical plot suggested publication bias [7].

## 3. Results

### 3.1. Preliminary Literature Screening Results

**Figure 1:**
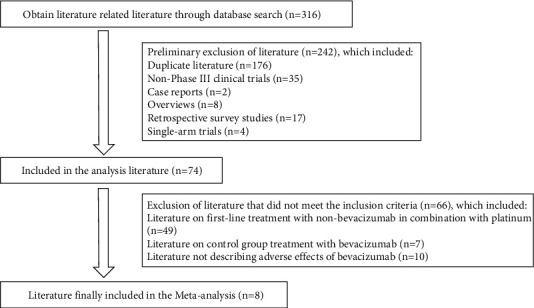
Preliminary literature screening results.

A total of 316 articles were initially screened, including 40 from PubMed, 168 from Embase, 44 from the Cochrane Library, 22 from CNKI, 24 from the Wanfang database, and 18 from VIP. Eight studies were finally included after excluding duplicate or ineligible literature, including seven studies on combined cisplatin perfusion [8, 10–15] and one on combined carboplatin perfusion [16], involving a total of 540 patients (271 cases in the trial group and 269 cases in the control group). The details are summarized in Figure 1.

### 3.2. Basic Characteristics of the Included Studies

Six of the included studies had been conducted on NSCLC patients and two on lung cancer patients. All patients were treated with thoracic perfusion. Bevacizumab and cisplatin perfusion were used in eight studies, and bevacizumab and carboplatin perfusion in one study, with cisplatin or carboplatin as the control group. The administered dosage of bevacizumab was 200 mg/dose in one study, 300 mg/dose in two studies, and 5 mg/kg in 6 studies (Table 1). In six studies, MPE was diagnosed by ultrasound, whereas two did not specify whether the diagnostic modality was CT or ultrasound. Nevertheless, the same diagnostic modality was used to compare pre- and posttreatment status in all studies.

### 3.3. Risk of Bias Assessment

**Figure 2:**
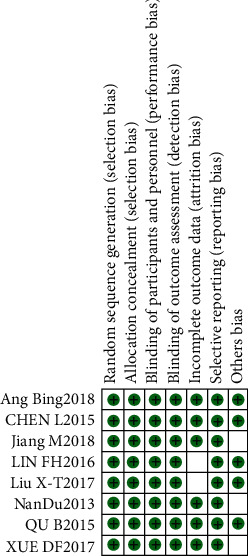
Risk of single-item bias in the included literature.

**Figure 3:**
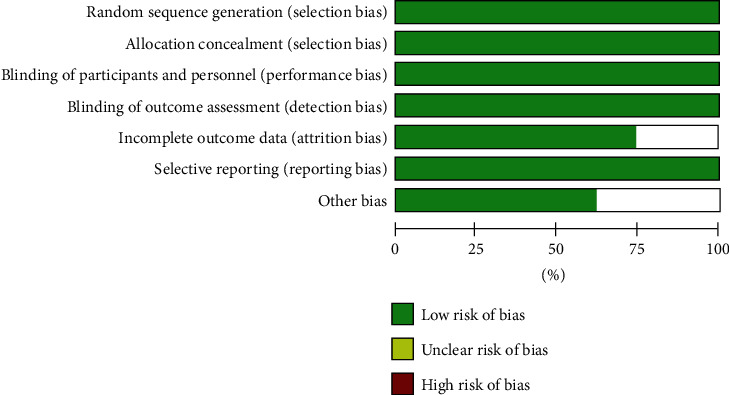
Overall risk of individual biases.

All eight studies showed a low risk of bias. The risk percentages of individual biases in each study are shown in Figure 2. The overall risk of individual biases is summarized in Figure 3.

### 3.4. Results of Meta-Analysis

#### 3.4.1. Rate of PR

**Figure 4:**
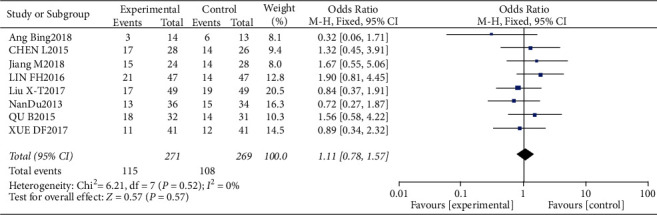
PR analysis.

**Figure 5:**
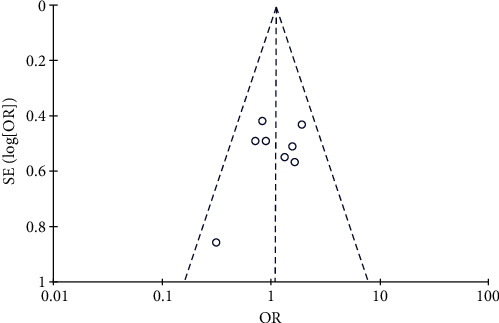
Results of funnel plot.

All studies included in the meta-analysis (271 cases in the trial group and 269 cases in the control group) reported PR. Due to low heterogeneity between the studies (*P*=0.52, *I*^2^ = 0), meta-analysis was conducted using the fixed-effects model. The rate of PR was higher in the trial group compared to the control group, albeit not statistically significant (OR = 1.11, 95% CI: 0.78–1.57, *P* > 0.05). The data are summarized in Figures 4 and 5.

#### 3.4.2. Rate of CR

**Figure 6:**
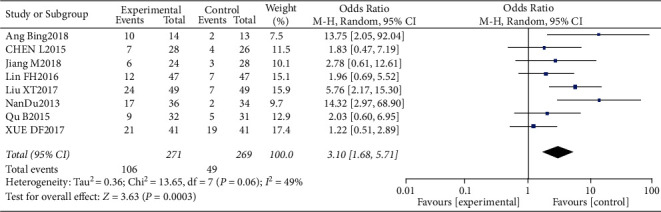
Results of CR analysis.

**Figure 7:**
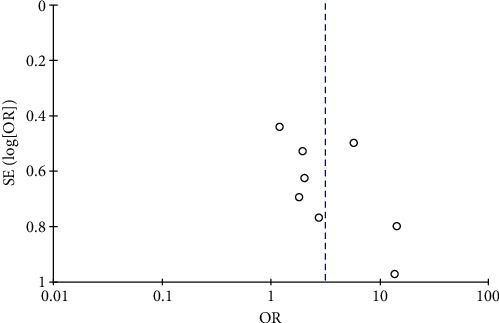
Results of funnel plots.

All studies included in the meta-analysis (271 cases in the trial group and 269 cases in the control group) reported CR. Statistical heterogeneity between the studies was significant (*P*=0.06, *I*^2^ = 49%), which warranted the random-effects model. The rate of CR was significantly higher in the test group compared to the control group (OR = 3.10, 95% CI: 1.68–5.71, *P* < 0.05). The data are shown in Figures 6 and 7.

#### 3.4.3. Risk of Leukopenia

**Figure 8:**
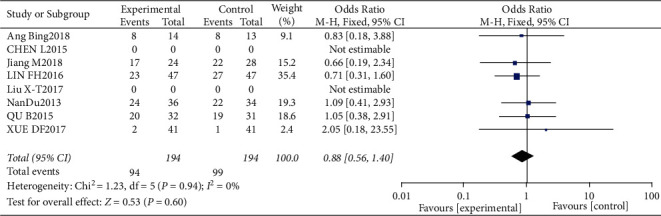
Results of leukopenia analysis.

**Figure 9:**
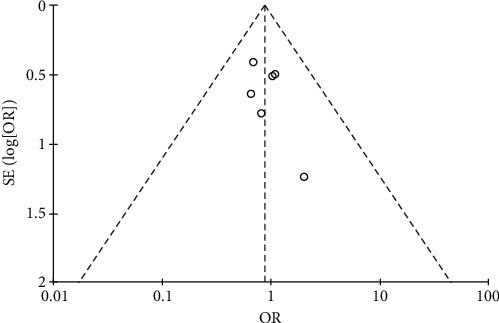
Results of funnel plots included in the literature.

Six studies, including 194 patients in the trial group and 194 patients in the control group, reported leucopenia. Statistical heterogeneity between the studies was low (*P*=0.94, *I*^2^ = 0), and the fixed-effects model was used. The risk of leukopenia was lower in the test group compared to the control group, although the difference was not statistically significant (OR = 0.88, 95% CI: 0.56–1.40, *P* > 0.05). The data are shown in Figures 8 and 9.

#### 3.4.4. Risk of Nausea and Vomiting

**Figure 10:**
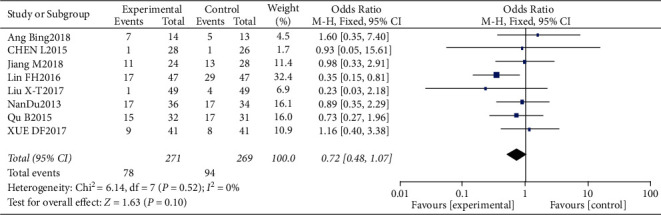
Results of nausea and vomiting analysis.

**Figure 11:**
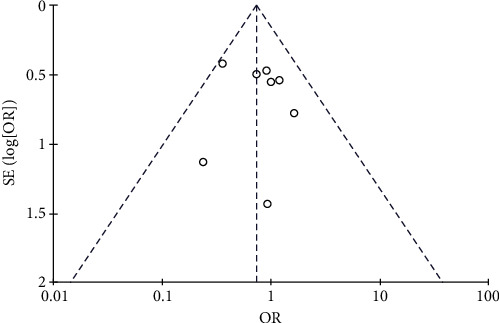
Results of funnel plots.

All studies reported the frequency of nausea and vomiting in the test and control groups. Statistical heterogeneity between the studies was low (*P*=0.52, *I*^2^ = 0), and the fixed-effects model was used. The test group had a slightly lower risk of nausea compared to the control group (OR = 0.72, 95% CI: 1.48–1.07, *P* > 0.05). The data are shown in Figures 10 and 11.

#### 3.4.5. Risk of Diarrhea

**Figure 12:**
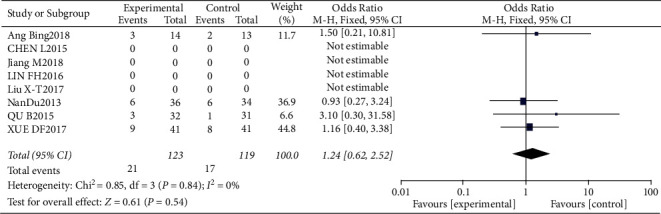
Results of diarrhea analysis.

**Figure 13:**
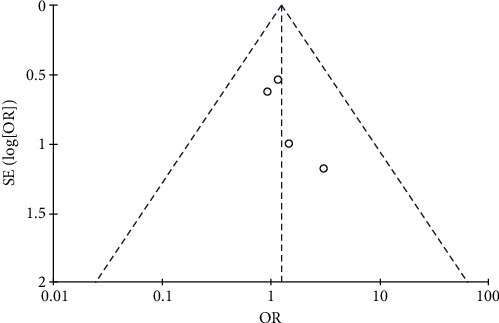
Results of funnel plots.

Diarrhea was reported in four studies that included 123 patients in the trial group and 119 patients in the control group. The fixed-effects model was used due to the low statistical heterogeneity between the studies (*P*=0.84, *I*^2^ = 0). The risk of diarrhea was higher in the test group compared to the control group, albeit without statistical significance (OR = 1.24, 95% CI: 0.62–2.52, *P* > 0.05). The data are shown in Figures 12 and 13.

#### 3.4.6. Risk of Nosebleeds, Hemoptysis, or Gastrointestinal Bleeding

**Figure 14:**
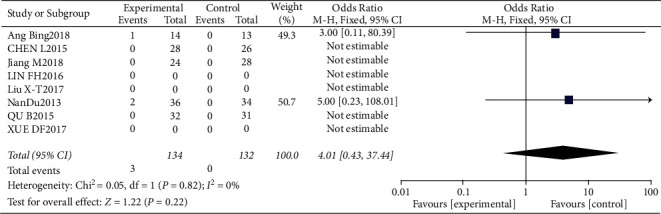
Results of the analysis of nosebleeds, hemoptysis, or gastrointestinal bleeding.

**Figure 15:**
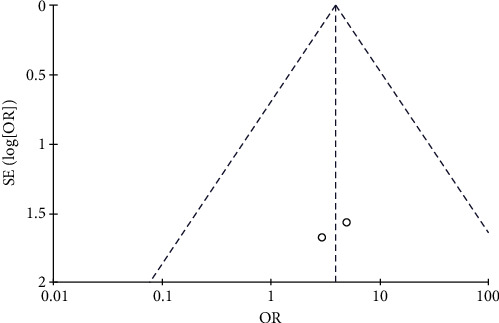
Results of funnel plots.

Five studies reported the frequency of nasal bleeding, hemoptysis, or gastrointestinal bleeding among 134 patients in the trial group and 132 patients in the control group. An increased risk of these events was observed in the trial group compared to the control group using the fixed-effects model (*P*=0.82, *I*^2^ = 0), although the difference was not statistically significant (OR = 4.01, 95% CI: 0.43–37.44, *P* > 0.05). The data are shown in Figures 14 and 15.

#### 3.4.7. Risk of Elevated Blood Pressure

**Figure 16:**
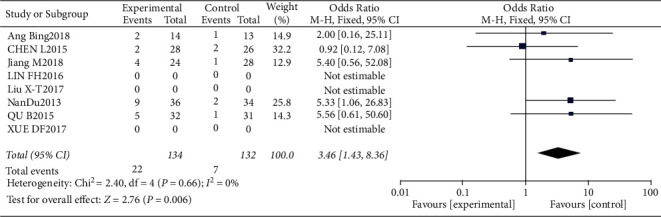
Results of the analysis of blood pressure elevation.

**Figure 17:**
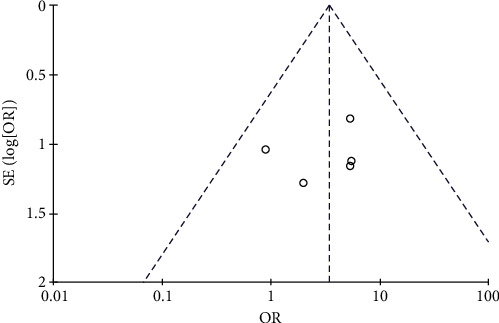
Results of funnel plots.

Five studies, including 134 patients in the trial group and 132 patients in the control group, reported elevated blood pressure. Statistical heterogeneity between the studies was low (*P*=0.66, *I*^2^ = 0), and the fixed-effects model was used. The risk of high blood pressure was significantly higher in the test group compared to the control group (OR = 3.46, 95% CI: 1.43–8.36, *P* < 0.05). The data are shown in Figures 16 and 17.

#### 3.4.8. Risk of Proteinuria

**Figure 18:**
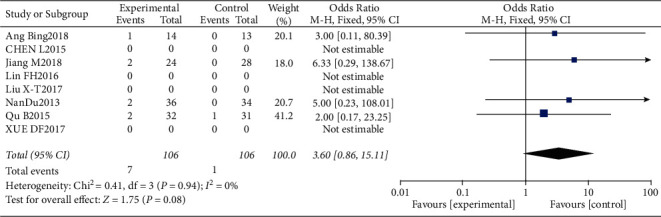
Results of proteinuria.

**Figure 19:**
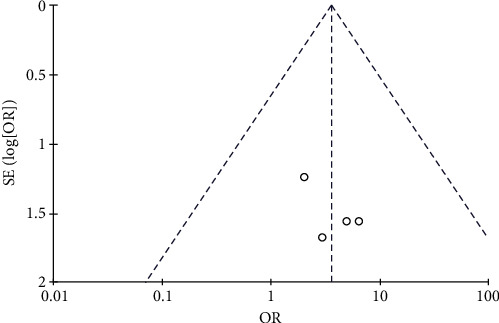
Results of funnel plots.

Four studies, including 106 patients in the trial group and 106 patients in the control group, reported proteinuria. Statistical heterogeneity between the studies was low (*P*=0.94, *I*^2^ = 0), and the fixed-effects model was used. The risk of proteinuria was higher in the test group compared to the control group, albeit not statistically significant (OR = 3.60, 95% CI: 0.86–15.11, *P* > 0.05). The data are shown in Figures 18 and 19.

#### 3.4.9. Incidence of Kidney and Liver Dysfunction

**Figure 20:**
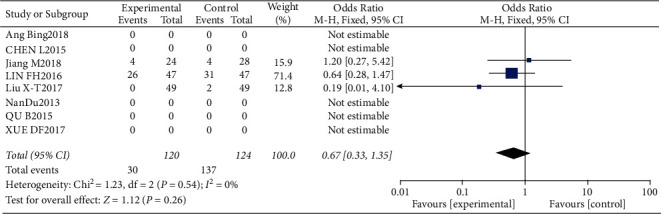
Results of the analysis of kidney and liver dysfunction.

**Figure 21:**
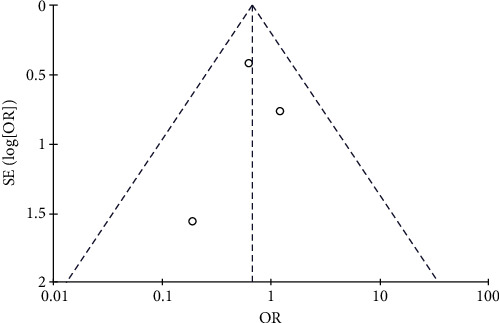
Results of funnel plots.

Three studies, including 120 patients in the trial group and 124 patients in the control group, reported abnormal liver and kidney function. The fixed-effects model was used on account of the low heterogeneity between the studies (*P*=0.54, *I*^2^ = 0). The patients in the test group showed a slightly lower risk of aberrant kidney and liver function compared to the control group (OR = 0.67, 95% CI: 0.33–1.35, *P* > 0.05). The data are shown in Figures 20 and 21.

#### 3.4.10. Risk of Arrhythmia

**Figure 22:**
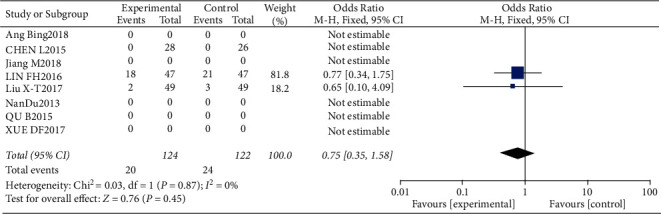
Results of arrhythmia.

**Figure 23:**
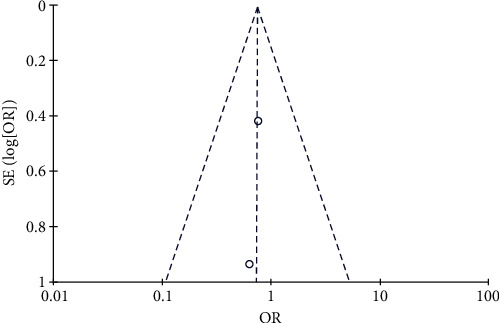
Results of funnel plots.

Three studies, including 124 patients in the trial group and 122 patients in the control group, reported arrhythmia. Statistical heterogeneity between the studies was low (*P*=0.87, *I*^2^ = 0), and the fixed-effects model was used. The risk of arrhythmia was slightly lower in the test group compared to the control group (OR = 0.75, 95% CI: 0.35–1.58, *P* > 0.05). The data are shown in Figures 22 and 23.

#### 3.4.11. Risk of Rashes

**Figure 24:**
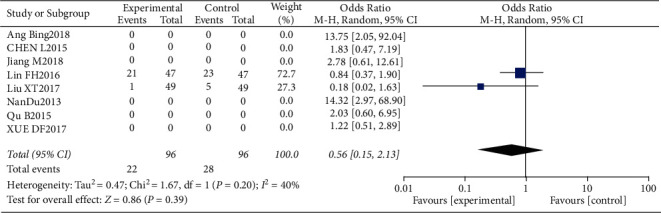
Results of rash analysis.

**Figure 25:**
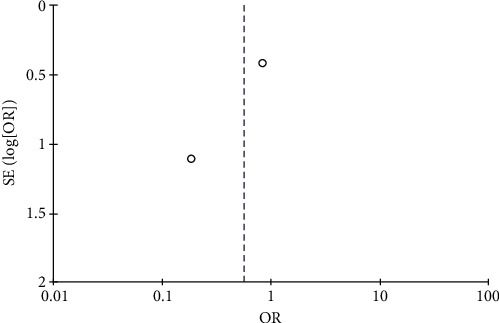
Results of funnel plots.

Two studies, including 96 patients in the trial group and 96 patients in the control group, reported an incidence of rashes. Since the statistical heterogeneity between the studies was significant (*P*=0.20, *I*^2^ = 40%), the random-effects model was used. Patients in the test group were at a slightly higher risk of developing rashes compared to the control group (OR = 0.56, 95% CI: 0.15–2.13, *P* > 0.05). The data are shown in Figures 24 and 25.

## 4. Discussion

MPE is a frequent complication of intestinal cancers, breast cancer, pleural mesothelioma, etc., and the highest incidence is observed in lung cancer patients (about 35%) [17]. The pathogenesis of MPE is complex. The key factors include lymphatic vessel obstruction, vascular endothelial cell damage, and increased vascular permeability, in addition to the decrease in plasma colloid osmotic pressure due to hypoproteinemia. However, the mechanisms through which tumor cells induce vascular damage are unclear.

Vascular endothelial growth factor (VEGF) promotes tumor neovascularization by increasing fibrinase production, which lyses the basement membrane and interstitial fibers of blood vessels, thereby encouraging the growth of new vessels. In addition, VEGF also participates in the formation of pleural effusion by malignant tumor cells [18–21]. Chen et al. found that VEGF competitively binds to receptors on endothelial cells and activates the mitogen-activated protein kinase signaling pathway, which induces their differentiation and promotes the formation of intercellular gaps, thereby increasing vascular permeability [22–26]. Li et al. reported that the significant increase in VEGF expression in MPE could distinguish the latter from benign pleural effusion, and treatment with bevacizumab led to VEGF blockade [6]. Therefore, VEGF is a key factor in MPE production and a predictive factor of its therapeutic regression.

MPE is routinely treated by thoracic infusion of chemotherapeutic drugs such as cisplatin, carboplatin, lopressor, and oxaliplatin, all of which are associated with systemic or local side effects and require multiple perfusions. Local perfusion of platinum drugs into the pleural cavity can alleviate MPE by directly killing the tumor cells and indirectly promoting the adhesion between the two layers of the pleura, which in turn inhibits MPE production. Although cisplatin and carboplatin have different pharmacokinetic characteristics, there is no significant difference in their therapeutic effects when administered intravenously. Studies comparing the effects of the intrathoracic/intrapleural instillation of cisplatin or carboplatin are limited. Xi et al. did not detect any significant difference between the therapeutic efficiency of intrathoracically instilled cisplatin and carboplatin. Xiaoyan et al. found that the therapeutic efficacy of cisplatin administered by pleural perfusion is only 50–60% [27], whereas Liang et al. reported 73.3% efficacy of similarly administered lobaplatin [28]. Therefore, local perfusion of platinum drugs is routinely combined with thymidine, Conrad injection, thermal perfusion therapy, interleukins, targeted drugs, etc., for treating MPE, and the combination therapies are superior to individual perfusion schemes in terms of efficacy and side effects. Lu et al. conducted a meta-analysis of eight RCTs, including a total of 328 patients, and found that thoracic perfusion of thymidine and oxaliplatin achieved greater efficacy against MPE compared to oxaliplatin alone, along with fewer side effects [29]. The majority of the studies included in the present meta-analysis showed that bevacizumab combined with platinum drugs was more effective than the individual drugs, albeit with a trend towards increased side effects compared to carboplatin alone. Nevertheless, it cannot yet be assumed that the dual drug combination increased the risk of adverse effects. Previous studies have also shown that the secondary increase in blood pressure due to bevacizumab is manageable and does not cause serious secondary damage.

Since VEGF is also essential for maintaining normal vascular endothelial cell function, blocking the VEGF signaling pathway can lead to endothelial dysfunction and hypertension. Several studies have shown that bevacizumab increases the risk of hypertension [30–32]. Therefore, blood pressure ≥150/95 mmHg before or during initial treatment warrants anti-hypertensive intervention and reevaluation of bevacizumab treatment after at least two weeks [33]. Amlodipine is recommended as the first choice for patients taking anti-hypertensive medications [33]. In addition, blood pressure measurement is recommended for patients prior to each administration of bevacizumab. Another concern of intrathoracic perfusion therapy is the extravasation of the perfused drugs into the subcutaneous tissues. Although it is a very rare occurrence, it is still necessary to verify the location of the intrathoracic tube before each infusion. Most studies included in this meta-analysis emphasized the need for multiple position changes after perfusion therapy to reduce the risk of drug extravasation and promote drug absorption.

To summarize, thoracic perfusion of bevacizumab combined with platinum-based drugs improves the survival and clinical outcomes of lung cancer patients with MPE without significantly increasing the risk of complications. However, the impact of this regimen on the long-term survival of MPE patients still needs to be further validated in a multicenter prospective study on a larger cohort.

The limitation of this meta-analysis is that we compared the effects of the combination treatment with carboplatin or cisplatin but not with bevacizumab due to the lack of data on bevacizumab as the control group. Therefore, more prospective studies should be done to analyze the difference between single-drug perfusion and multiple-drug perfusion therapy or between single-drug perfusion and thoracic fever perfusion. Recent meta-analyses suggest that intravenous and intrathoracic administration of bevacizumab have the same efficacy, whereas our data indicate that chest infusion has greater therapeutic benefit.

## 5. Conclusion

Bevacizumab combined with platinum-based thoracic perfusion can achieve CR of MPE in patients with advanced lung cancer without significantly increasing the risk of adverse effects. The rate of PR was similar for the combination treatment and platinum-based infusion.

## Figures and Tables

**Table 1:** Basic characteristics of the included studies.

Inclusion in studies	Country	Type of pathology	Staging	Total number of cases	Number of cases in each group		Gender (male/female)	Number of treatment lines	Interventions		Evaluation indicators
					Experimental group	Control group			Experimental group	Control group	
Nan Du 2013	China	Non-small-cell lung cancer	IV	70	36	34	39/32	First-line treatment	Cisplatin (60 mg) + bevacizumab (300 mg) dissolved in saline 20 ml by intrathoracic infusion, every 2 weeks, at least two courses of treatment	Cisplatin (60 mg) dissolved in saline 20 ml for intrathoracic instillation	①, ②, ③, ④, ⑤, ⑥, ⑦, ⑧
Liu X.-T. 2017	China	Non-small-cell lung cancer	IV	98	49	49	54/44	First-line treatment	Bevacizumab (5 mg/kg) + cisplatin (45 mg/m^2^) was dissolved in 20 mg of 0.9% sodium chloride and injected intrathoracically through a central venous catheter once/week for 3 weeks	Cisplatin (45 mg/m^2^) dissolved in 20 mg 0.9% sodium chloride for intravenous injection	①, ②, ④, ⑨, ⑩,
Jiang M. 2018	China	Non-small-cell lung cancer	IV	52	24	28	33/19	First-line treatment	Bevacizumab (5 mg/kg) + saline (20 mL) was administered once every 7 days via central venous catheter by thoracic infusion for 3 consecutive doses	Carboplatin injection (300 mg) + saline (50 mL) via central venous catheter for thoracic infusion	①, ②, ③, ④, ⑥, ⑦, ⑧, ⑨,
Ang Bing 2018	China	Non-small-cell lung cancer	IV	27	14	13	15/12	First-line treatment	Bevacizumab (200 mg) + cisplatin (60 mg/m^2^) by intrathoracic infusion, d1, 2 times/week for 3 weeks	Cisplatin (60 mg/m^2^) by intrathoracic infusion	①, ②, ③, ④, ⑤, ⑥, ⑦, ⑧
Qu B. 2015	China	Non-small-cell lung cancer	IV	63	32	31	36/27	First-line treatment	Bevacizumab (5 mg/kg) + cisplatin (40 mg/m^2^) by intrathoracic infusion, 1 time/week, 3 cycles	Cisplatin (40 mg/m^2^) by intrathoracic infusion	(①, ②, ③, ④, ⑤, ⑥, ⑦, ⑧
Lin F. H. 2016	China	Non-small-cell lung cancer	IV	94	47	47	50/44	First-line treatment	Bevacizumab (5 mg/kg) + cisplatin (45 mg/m^2^), dissolved in 20 ml of saline and injected into the chest cavity through a central venous catheter once a week for 3 weeks	Cisplatin (45 mg/m^2^) dissolved in 20 ml saline for intrathoracic instillation	①, ②, ③, ④, ⑨, ⑩, ⑪
Xue D. F. 2017	China	Non-small-cell lung cancer	IV	82	41	41	47/35	First-line treatment	Bevacizumab (5 mg/kg) + cisplatin (60 mg) by intrathoracic infusion, once a week for 3 weeks	Cisplatin (60 mg) by intrathoracic infusion	(①, ②, ③, ④, ⑤
Chen L. 2015	China	Non-small-cell lung cancer	IV	54	28	26	27/17	First-line treatment	Bevacizumab, (5 mg/kg) + cisplatin (75 mg/m^2^) by intrathoracic infusion, divided into 2 cycles of 21 d each	Cisplatin (75 mg/m^2^) by intrathoracic infusion	①, ②, ④, ⑦, ⑧, ⑩

*Note.* ① CR, complete remission; ② PR, partial remission; ③ leukopenia; ④ nausea and vomiting; ⑤ diarrhea; ⑥ rhinorrhea; hemoptysis or gastrointestinal bleeding; ⑦ elevated blood pressure; ⑧ proteinuria; ⑨ abnormal liver and kidney function; ⑩ heart rate disorders; ⑪ skin rash.

## Data Availability

All data generated and analyzed in this study are included in this published article and are available upon request.
